# Spontaneous cooperation for prosocials, but not for proselfs: Social value orientation moderates spontaneous cooperation behavior

**DOI:** 10.1038/srep21555

**Published:** 2016-02-15

**Authors:** Dorothee Mischkowski, Andreas Glöckner

**Affiliations:** 1 University of Göttingen, Department of Psychology, Gosslerstrasse 14, D-37073 Göttingen, Germany; 2 University of Hagen, Department of Psychology, Universitaetsstrasse 27, D-58097 Hagen, Germany; 3 Max Planck Institute for Research on Collective Goods, Kurt-Schumacher-Str. 10, D-53113 Bonn, Germany.

## Abstract

Cooperation is essential for the success of societies and there is an ongoing debate whether individuals have therefore developed a general spontaneous tendency to cooperate or not. Findings that cooperative behavior is related to shorter decision times provide support for the spontaneous cooperation effect, although contrary results have also been reported. We show that cooperative behavior is better described as person × situation interaction, in that there is a spontaneous cooperation effect for prosocial but not for proself persons. In three studies, one involving population representative samples from the US and Germany, we found that cooperation in a public good game is dependent on an interaction between individuals’ social value orientation and decision time. Increasing deliberation about the dilemma situation does not affect persons that are selfish to begin with, but it is related to decreasing cooperation for prosocial persons that gain positive utility from outcomes of others and score high on the related general personality trait honesty/humility. Our results demonstrate that the spontaneous cooperation hypothesis has to be qualified in that it is limited to persons with a specific personality and social values. Furthermore, they allow reconciling conflicting previous findings by identifying an important moderator for the effect.

Cooperation is important for societies to achieve collective goals^1^. In order to be able to foster cooperation a detailed understanding of the processes underlying individuals’ decisions to cooperate (or not) is essential and research has increasingly focused on investigating these cognitive processes in more detail^2^,^3^,^4^,^5^. One core matter of debate has been whether individuals have a spontaneous tendency to cooperate and findings both in favor^3^,^4^,^6^ and in conflict^2^,^7^,^8^ with this hypothesis have been reported. This inconsistent evidence indicates that important moderators that might lie in the situation, the person or their interaction have not been identified yet.

Previous experience with the experimental setting of social dilemma games has been found to reduce the effect^4^, and on theoretical^9^ and empirical^10^,^11^ grounds it can be expected that other situational factors that influence the activation of social norms such as situation framing affect spontaneous behavior as well. Tests for effects of individual difference moderators have (aside from several null findings) revealed that the spontaneous cooperation effect is observable mainly for persons trusting in the cooperativeness of their daily life interaction partners and it is not observed for persons with experience in related studies^4^. Still a comprehensive analysis of personality moderators is largely missing.

We guide the perspective towards social value orientation (SVO), which is a continuous measure of social preferences and can be expected to be an important moderator for the spontaneous cooperation hypothesis on theoretical grounds^3^,^12^,^13^. Specifically, we hypothesize that the spontaneous cooperation effect might be driven by prosocials–having a default willingness to cooperate, which tends to disappear with longer deliberation about the dilemma structure of the situation. No effect of time is expected for proself persons. Based on the findings concerning spontaneous cooperation and taking into account that SVO has been established as a strong predictor of cooperation^14^,^15^, we expect main effects for SVO, decision time and–most importantly–their interaction on cooperation behavior.

## Method

We conducted three studies including one lab and two online experiments. The studies were approved by the local ethics committee of Göttingen University’s Psychological Department and conducted in accordance with the approved guidelines. Before conducting the studies, we obtained informed consent from all subjects. With the methods and instructions used, we strictly replicated the previous core investigation by Rand and colleagues^4^, except for some important extensions. Participants played one-shot public good games (PGG) in groups of four. In the PGG, independent and anonymous individual contributions of each member were doubled and split evenly among the members of the group.

**Figure 1:**
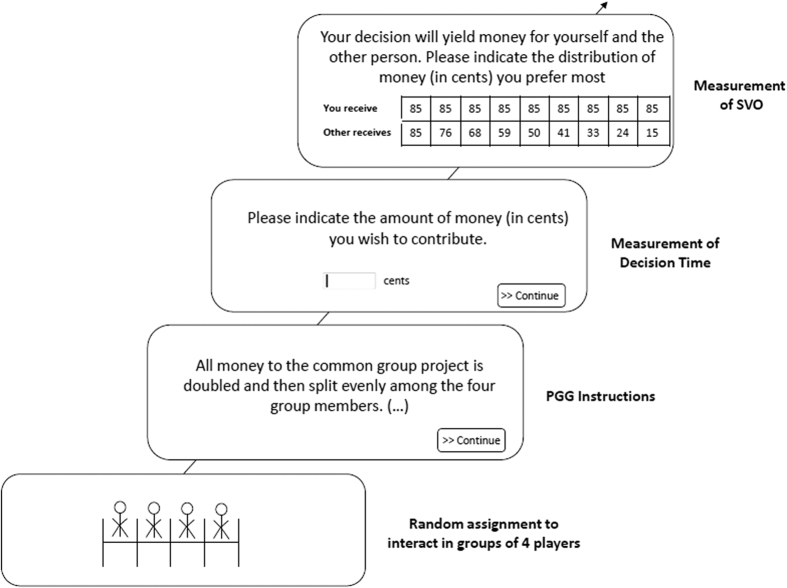
Procedure of the studies. Note: PGG refers to Public Goods Game, SVO stands for Social Value Orientation.

The basic procedure is described in Fig. 1. After group assignment, participants were presented with an instruction screen describing the structure and the rules of the game. After reading the screen, participants clicked a continue button to proceed to the response screen, which contained the request to indicate the amount of money (in cents) they wish to contribute. Participants indicated their contributions by typing a number in the respective field and confirmed their input with a mouse click on a continue button. The time between onset of the response screen and the confirmation of the contribution by mouse click constituted our main predictor variable decision time. Hence, decision time exclusively captures the time required for generating the response concerning how much to contribute. The reading time of the Public Goods Game instructions was explicitly detached by displaying instruction on a separate screen.

Decision time was measured via the programs used for data collection (i.e.‘Unipark’ for the online studies and ‘Bonn Experiment System’ for the lab study). In the first study we analyzed data of 134 Amazon Mechanical Turk (MTurk) workers (51 female, mean age = 30.5 years) half of them from the United States and the remaining from other western countries and India. Second, we elaborated these results in a more controlled setting and conducted a laboratory study with mostly German students (N = 105, 70 female, mean age = 26.97 years). Finally, as a third experiment, we conducted a high-power online study with representative samples concerning age and gender for the US (N = 249) and German populations (N = 255), resulting in a total sample of N = 504 (258 female, mean age = 46.13 years). Data for the third experiment was gathered via a professional panel provider. The endowment for the PGG varied between studies (i.e., USD 0.40 in the first study conducted on MTurk, 2.00€ (approx. USD 2.70) in the lab experiment and USD 1.50 in the third study (Panel)) but the multiplication factor of two for contributed money remained constant.

In line with previous studies we additionally gathered data about beliefs (the expectations about the other players’ cooperation behavior) as well as cooperativeness of interaction partners in daily life and previous experience with the experimental setting of social dilemma games. As an important extension, we additionally measured social value orientation using the SVO Slider Measure^16^ at the end of the study, which allows to calculate a continuous SVO angle value. An angle of around zero indicates proself persons; these are persons that maximize their own outcomes without considering outcomes of others. Positive values indicate more prosocial behavior in that people gain positive utility from outcomes of others. The SVO measure was incentivized and it was common knowledge that it was determined by a random draw whether the PGG or the SVO were incentivized. In order to check for correlations with broader personality factors, we included the 60 items general personality questionnaire HEXACO at the end of the third study^17^. More details on the procedure and the fully instruction are provided in the supplementary online materials.

## Results

In an overall analysis of the three experiments (Table 1), we find support for the spontaneous cooperation hypothesis in that contributions increase with decreasing decision time (see Fig. S1). Increasing decision times from 3.16 sec (10^0.5^ = 3.16) to 31.6 sec (10^1.5^ = 31.6) was accompanied by a decrease of cooperation by 54.52% according to a Tobit regression and by 19.08% according to an OLS regression (see Table S2). Considering the three studies separately, we replicate the spontaneous cooperation main effect in Study 3 (the representative online sample study) and find trends in the same direction for the other two studies with lower power. As a side effect, one should note that there are roughly 10% differences in the average contributions among studies, being highest in the panel sample (67.10%) and lowest in the MTurk sample (44.59%; one-way ANOVA: *F*(2, 740) = 17.21, *p* < 0.001). This difference was paralleled by a difference in social value orientation (one-way ANOVA: *F*(2, 740) = 30.68, *p* < 0.001) with around 5° of SVO angle differences between studies. The parallel development of contributions and SVO is–as expected–reflected in a strong correlation between both measures (*r* = 0.48, *p* < 0.001). Hence, differences in social values (social preferences) among the different samples used in our studies have most likely driven the level differences in contributions. To correct for these differences, we included study dummies as control variables in the overall analysis of contributions.

**Figure 2:**
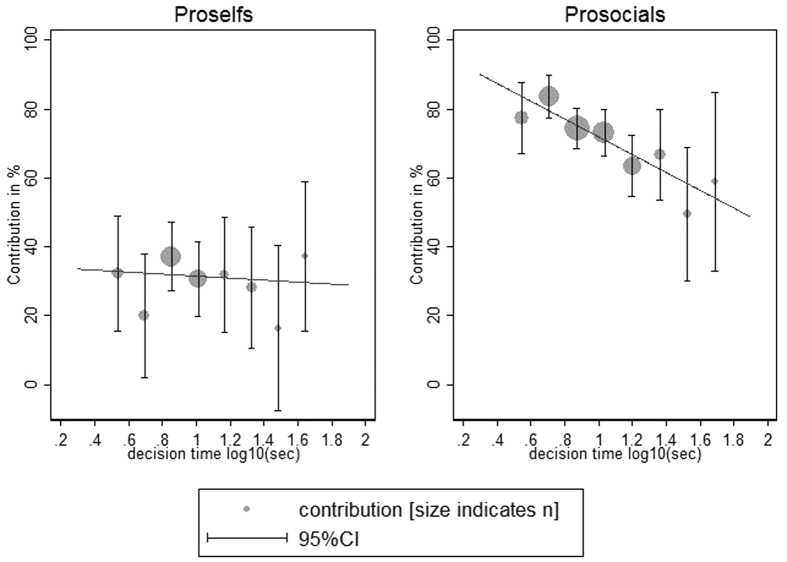
There is a spontaneous cooperation effect for prosocials (SVO angle >22.45°), but not for proself persons (SVO angle <22.45°). Prosocials contribute less the longer the decision time (decision time between 2 and 72 seconds). For proselfs, however, there is no difference in cooperation behavior, independent of decision time they stick to low contributions. Regression lines represent the effect of reaction times on contributions from separate OLS regressions. Whiskers denote the 95% confidence interval; the diameter of the grey dots is proportional to the sample size.

Most importantly, in this overall analysis we find the expected interaction of decision time and SVO (Fig. 2). In the separate analyses comparable effects are found for all three studies although in the MTurk study the effect fails to reach conventional levels of significance.

Separate analyses for prosocials (SVO angle > 22.45°) and proselfs (SVO angle < 22.45°) reveal that the spontaneous cooperation effect is only observed for prosocials (Tobit and OLS overall: *p* < 0.001) but does not hold for proselfs (Tobit: *p* = 0.836; OLS: *p* = 0.475). This also holds true in separate analyses for the three studies (see Table S3). Furthermore, the observed interaction also holds when controlling for the previously observed moderators cooperativeness of daily life interaction partners and experience in related studies (Table S4).

To validate our results also on the basis of a broader personality construct, we measured Honesty-Humility as one of the six factor personality scale HEXACO^17^,^18^. Honesty-Humility represents “the tendency to be fair and genuine in dealing with others, in the sense of cooperating with others even when one might exploit them without suffering retaliation” (p.156)^18^ and has shown to be highly predictive of cooperation behavior^19^,^20^. Again, we find the same interaction pattern in that cooperation decreases with decision time for people high on Honesty-Humility only but not for people low in this personality trait (Table S5). As in previous studies, SVO and Honesty-Humility are moderately correlated (*r* = 0.22, *p* < 0.001), but the interaction effect persists on a marginal significance level also when controlling for SVO and its interaction with decision time (*p* = 0.064). The observed interaction concerning the specific construct social values therefore also generalizes to the related but broader personality construct Honesty-Humility.

## Discussion

In a set of studies we show that the controversially debated spontaneous cooperation effect replicates in population representative samples and beyond. Given its small effect size, large samples are needed in order to find the effect on a conventional significance level. Crucially, however, we show that the spontaneous cooperation effect is also strongly dependent on personal characteristics. For persons that are prosocial and high on the personality factor Honesty-Humility cooperation decreased with increasing decision time. These persons drive the spontaneous cooperation effect reported in some of the previous studies. No relation between decision time and cooperation exists for selfish persons. The demonstration of this moderator allows reconciling previous diverging results.

The finding that only prosocials show the spontaneous cooperation effect is consistent with the explanation that cooperation might be their default response, which is sometimes overwritten by increased deliberation. Given that prosocials are mainly conditional cooperators that are particularly sensitive to changes in beliefs concerning the behavior of other group members^21^ deliberate reflection about the game structure might decrease their contributions. No such adjustments can be expected for proself persons. Still, given the correlational design of our research such causal interpretations of processes cannot be directly tested, which is due to further research.

## Additional Information

**Data Availability:** Data and materials are also available online at Open Science Framework (https://osf.io/w7hsk/).

**How to cite this article**: Mischkowski, D. and Glöckner, A. Spontaneous cooperation for prosocials, but not for proselfs: Social value orientation moderates spontaneous cooperation behavior. *Sci. Rep.* **6**, 21555; doi: 10.1038/srep21555 (2016).

## Supplementary Material

Supplementary Information

## Figures and Tables

**Table 1:** Tobit regression of decision times and Social Value Orientation on contributions.

Contribution (in %)	Overall Analysis	Study 1 (MTurk)	Study 2 (Lab)	Study 3 (Panel)
Decision Time(DT in log10(sec))	−54.52^***^(−3.94)	−25.64(−1.20)	−42.78(−1.02)	−64.76^**^(−3.48)
Social Value Orientation(SVO angle in degree)	3.19^***^(11.39)	3.86^***^(6.80)	2.71^***^(3.94)	3.00^***^(8.39)
Interaction of DT * SVO	−2.90^**^(−3.19)	−2.14(−1.40)	−4.80^+^(−1.77)	−2.52^*^(−1.98)
Constant	66.37^***^(7.59)	42.23^***^(5.81)	75.05^***^(7.37)	100.74^***^(19.25)
Observations	743	134	105	504
Pseudo R2	0.052	0.083	0.041	0.036

^+^p < 0.1, *p < 0.05, **p < 0.01, ***p < 0.001 (two-sided). Note. *t*-values are presented in parentheses; predictors are centered. Overall analysis also includes two study dummies which are not reported.
