# Supplementary material for: Spontaneous cooperation for prosocials, but not for proselfs: Social value orientation moderates spontaneous cooperation behavior
Source: Sci Rep. 2016 Feb 15;6:21555. doi: 10.1038/srep21555 (PMC4753511; doi:10.1038/srep21555)
Supplement: Supplementary Information [file srep21555-s1.pdf]

Supplementary Materials

Spontaneous cooperation for prosocials, but not for proselfs:

Social value orientation moderates spontaneous cooperation behavior

Dorothee Mischkowski[1] & Andreas Glöckner*[2,3]

[1] University of Göttingen

[2] University of Hagen

[3] Max Planck Institute for Research on Collective Goods, Bonn

*Correspondence should be addressed to: Andreas Glöckner, University of Hagen, Universitaetsstrasse 27, D-58097 Hagen, Germany, Phone: +49-2331 987 2180, E-mail: andreas.gloeckner@fernuni-hagen.de

1. Sample characteristics

The first study was conducted on Amazon Mechanical Turk (MTurk) to stick as closely as possible to the methods used by Rand, Greene, and Nowak (2012)[1]. The sample consists of 134 MTurk workers (51 female) with a mean age of 30.5 years (range 18-62 years), half of them from the United States and the remaining from other western countries and India. The second study collected data of 105 participants (70 female participants), mainly German students with mean age of 26.97 years (range 19-62 years). The representative Panel sample for US and German population consists of 504 participants in total (258 female) and a mean age of 46.13 years (range 18-77 years). The sub-sample of US citizens consisted of 249 participants, the German subsample accordingly of 255 participants with similar age and gender distributions. We excluded 4 participants in Study 1, 4 participants in Study 2 and 11 participants in Study 3 due to too long reaction times (more than + 3 SD from the mean), lack of game comprehension (contributions > 100%), or as drop-out during the study. Including them did not change the results in the overall analysis and only slightly but unsystematically in the separate analyses (i.e. for the better in Study 1 and for worse in Study 3). Furthermore, participants who answered control questions about the game structure incorrectly were screened out and not allowed to take part in the studies.

2. Method and experimental instructions

We used the instructions of Rand et al.[1] in Study 1 and for the US subsample in Study 3. The same instruction was translated into German or the Study 2 and the German subsample in Study 3. In the first two studies we additionally manipulated the framing of the game as either ‘Wall Street Game’ or ‘Community Game’[2]. This manipulation does not influence the presented results, which persist when including the framing as a covariate.

First participants were introduced to the setting before being familiarized with the instruction of the Public Good Game (PGG). The decision how much to contribute was displayed on a new page in order to disentangle reading time from decision time. Control questions assured the understanding of the game structure and were asked afterwards, in order not to prime any reflection beforehand. A different order was used in Study 2 (lab study), in which participants answered the questions at the very beginning of the experiment. This was done in order to avoid a costly dropout of participants. In the online studies, participants with incorrect understanding of the game structure were automatically screened out by a filter option.

We measured beliefs after the control questions, in order to avoid biased contributions by deliberation taken beforehand. The next screen asks for the cooperativeness of daily life interaction partners, identified as a moderator of spontaneous cooperation by Rand et al. (2012). Cooperativeness is operationalized via trust in order to assure a uniform and more familiar understanding of cooperativeness[3]. Afterwards, we extended the replication of Rand et al. (2012) by including the Social Value Orientation (SVO) Slider Measure of Murphy, Ackermann, and Handgraaf [4]. Participants were clearly instructed that either the PGG or the SVO Slider would randomly be chosen in order to calculate the bonus payment. Both tasks offered roughly the same expected value.

In the panel study, the HEXACO-6 factor model of personality was included afterwards, before an exploratory instructional manipulation check[5] that was mainly included to make inferences about the framing manipulation in the first to studies as either ‘Wall Street Game’ or ‘Community Game’. We don’t pursue this manipulation in this paper any further, but (as mentioned above) our results persist

when including the framing as a covariate. Finally, previous experience with the experimental setting of social dilemma games and demographic variables was enquired.

Instructions of the Panel study

Screen 1 (Introduction)

Thank you for taking part in this study. You will receive a basic incentive. In addition, you have the opportunity to receive additionally up to $3.75 for one of the two major tasks this study consists of. Both offer you about the same amount of bonus payment. It is important that you answer both tasks; one will be randomly selected to calculate your payment.

Screen 2 (PGG instructions)

You have been randomly assigned to interact with 3 other people. All of you receive this same set of instructions. You cannot participate in this study more than once.
Each person in your group is given 150 cents for this interaction (in addition to the basic incentive you already received for participating).
You decide how much of your 150 cents to keep for yourself, and how much (if any) to contribute to the group's common project (in increments of 2 units: 0, 2, 4, 6 cents etc).
All money to the common project is doubled, and then split evenly among the 4 group members.
Thus, for every 2 cents contributed to the common project, each group member receives 1 cent.
If everyone contributes all of their 150 cents, everyone's money will double: each of you will earn 300 cents.

But if everyone contributes their 150 cents while you keep your 150 cents, you will earn 375 cents, while the others will earn only 225 cents. That is because for every 2 cents you contribute, you get only 1 cent back. Thus, you personally lose money on contributing.
The other people are REAL and will really make a decision - there is no deception in this study.
Once you and the other people have chosen how much to contribute, the interaction is over. Neither you nor the other people receive any bonus other than what comes out of this interaction (in case this task is chosen to calculate your bonus payment).

Screen 3 (Contribution)

Please indicate the amount of money (in cents) you wish to contribute.

Screen 4 (Control question I)

What level of contribution (in cents) earns the highest payoff for **the group as a whole**?

Screen 5 (Control question II)

What level of contribution (in cents) earns the highest payoff for you **personally**?

Screen 6 (Beliefs)

What do you think will be the average contribution (in cents) of the others to the common project?

Screen 7 (Cooperativeness of interaction partners in daily life)

To what extent do you feel you can trust other people that you interact with in your daily life?

Screen 8 (SVO instructions)

This is the second task; it is not related to the first one. Here, you have been randomly paired with another person, one who has not been in the group before. We will refer to him or her as the other. This other person is someone you do not know and will remain mutually anonymous. All of your choices are completely confidential. You will be making a series of decisions about allocating resources between you and this other person. One choice is randomly selected and payed as bonus fee for you and the other. For each of the following questions, please indicate the distribution of money you prefer most by **marking the respective position along the midline**. You can only make one mark for each question.
Your decisions will yield money for both yourself and the other person. The monetary unit of the displayed numbers is cent. In the example below, a person has chosen to distribute money so that he/she receives 50 cents, while the anonymous other person receives 40 cents.

There are no right or wrong answers; this is all about personal preferences. As you can see, your choices will influence both the amount of money you receive as well as the amount of money the other receives (in case this task is chosen to calculate your bonus payment).

Screen 9 (SVO Slider Measure)

SVO Slider Measure (6 items) [4]

Screen 10 (HEXACO)

HEXACO-6-factor model of personality (60 items) [6]

Screen 11 (Instructional manipulation check)

Modern theories about decision making processes have shown that decisions are influenced by many different aspects. Individual preferences, prior knowledge and context variables influence the decision process. To improve our research on decision making, we are interested in getting to know some aspects about you, the decision maker. Especially, we are interested if you take enough time to read the instructions carefully. If you don't, some of our manipulations based on differences in the instructions are ineffective. This is why we ask you to ignore the question at the bottom of this page and to click on all response options instead in order to show us that you read the instructions. Afterwards, you can proceed to the next page. Thank you very much!

Which of these countries have you already been to? Please tick in as appropriate.

Screen 12 (Previous experience)

To what extent have you participated in studies like this one before? (i.e. studies in which you choose how much to keep for yourself versus contributing to benefit others)

3. Results

Table S1 presents descriptive statistics, summarizing demographics and the main independent and dependent variables.

Table S1. Descriptive statistics

	Mean (Std)		
	**Study 1 (MTurk)**	**Study 2 (Lab)**	**Study 3 (Panel)**
**Contribution (in %)**	44.59 (42.15)	55.95 (43.84)	67.10 (39.81)
**Decision Time (in sec)**	11.60 (11.68)	15.64 (9.23)	11.20 (10.30)
**Log10 (Decision Time)**	0.92 (0.34)	1.13 (0.23)	0.96 (0.25)
**Social Value Orientation (SVO angle in degree)**	20.23 (15.56)	25.93 (16.22)	31.01 (13.92)
**Age**	30.50 (9.87)	26.97 (7.58)	46.13 (14.90)
**Gender (in % female)**	38	67	51
**US Residency (in %)**	50	-	49
**N**	134	105	504

Note. Log10 Decision time represents logarithmized decision times to the base 10.

Note that we observed considerable differences concerning the level of decision time between studies, with participants in Study 2 taking much longer than in the other studies (S1). This could be due to the fact that participants in the lab (Study 2) might generally take more time to decide in comparison to online samples (Study 1 and 3). The payment in the lab is usually higher than that of the online providers so that people might be used to work more carefully. Furthermore, it might be more tempting to increase hourly wages by shortening the time to work on a task in an anonymous online setting as compared to a lab involving some aspects of group norms.

Comparing the results of the Tobit regression presented in Table 1 to an OLS regression, the same general pattern of results holds true (Table S2).

Table S2. OLS regression of decision times and SVO angle on contributions

**Contribution (in %)**	**Overall Analysis**	**Study 1 (MTurk)**	**Study 2 (Lab)**	**Study 3 (Panel)**
**Decision Time (DT in log10(sec))**	-19.08*** (-3.58)	-11.10 (-1.23)	-17.53 (-0.89)	-21.05** (-3.08)
**Social Value Orientation (SVO angle in degree)**	1.24*** (13.58)	1.70*** (10.11)	0.93*** (3.74)	1.17*** (9.96)
**Interaction DT * SVO**	-0.86* (-2.59)	-0.92 (-1.45)	-1.66 (-1.41)	-0.46 (-1.08)
**Constant**	53.98*** (17.88)	44.56*** (15.84)	56.01*** (14.07)	67.11*** (41.82)
**Observations**	743	134	105	504
**Adjusted R^2**	0.248	0.405	0.138	0.180

+ p < .1, * p < .05, ** p < .01, *** p < .001 (two-sided)

Note. *t*-values are presented in parentheses; predictors are centered. Overall analysis also includes two study dummies which are not reported.

Figure S1 shows that the overall spontaneous cooperation effect replicating Rand et al., 2012.

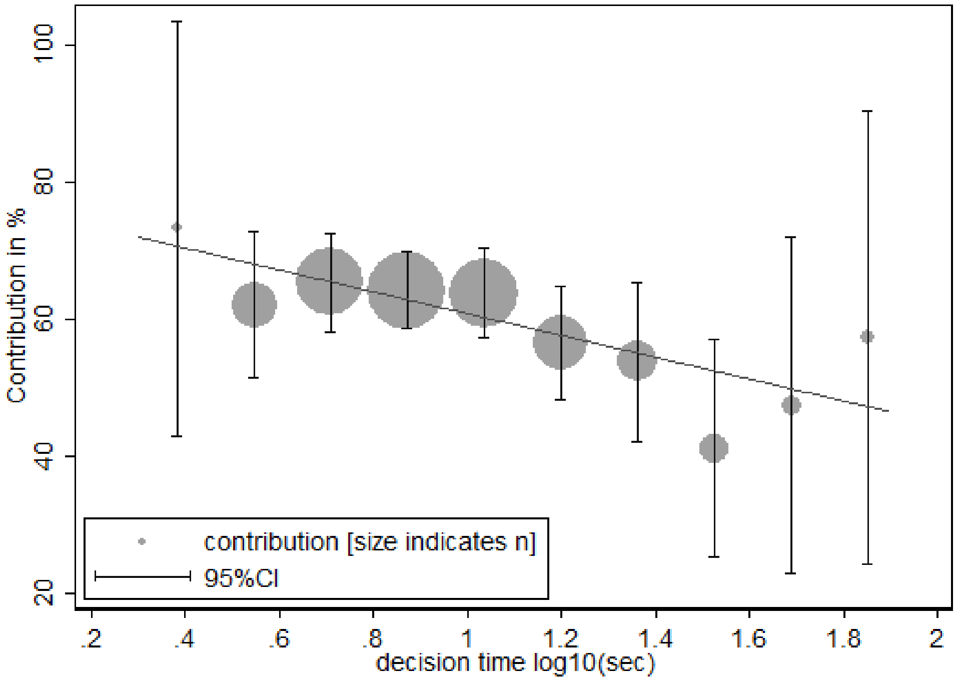

Figure S1: Replication of the spontaneous cooperation effect over all three studies (N=743). Regression line represents the effect of reaction times on contributions from an OLS regression. Whiskers denote the 95% confidence interval; the diameter of the grey dots is proportional to the sample size.

When splitting the sample in proselfs (SVO angle < 22.45°) versus prosocials (SVO angle > 22.45°), the spontaneous cooperation effect can only be found for prosocials (Table S3).

Table S3. Tobit regression of decision time on contributions separately for types of SVO angle

Prosocials (SVO angle > 22.45°)

Contribution (in %)	Overall Analysis	Study 1 (MTurk)	Study 2 (Lab)	Study 3 (Panel)
**Decision Time (DT in log10(sec))**	-73.05*** (-4.18)	-70.08* (-2.15)	-90.99 (-1.64)	-70.61** (-3.22)
**Constant**	174.20*** (8.05)	168.60*** (4.72)	205.18** (3.07)	186.73*** (8.11)
**Observations**	538	66	72	400
**Pseudo R^2**	0.008	0.014	0.007	0.006

+ p < .1, * p < .05, ** p < .01, *** p < .001 (two-sided)

Proselfs (SVO angle < 22.45°)

Contribution (in %)	Overall Analysis	Study 1 (MTurk)	Study 2 (Lab)	Study 3 (Panel)
**Decision Time (DT in log10(sec))**	-4.79 (-0.21)	-0.81 (-0.03)	31.27 (0.46)	-16.59 (-0.47)
**Constant**	-12.77 (-0.52)	-8.83 (-0.29)	-15.13 (-0.19)	45.82 (1.29)
**Observations**	205	68	33	104
**Pseudo R^2**	0.009	0.000	0.001	0.000

Note. *t*-values are presented in parentheses. Overall analysis also includes two study dummies which are not reported.

Further analyses revealed that the moderating effect of SVO angle with regard to the spontaneous cooperation effect goes beyond the effects of previously observed moderators (Table S4). The coefficient of the SVO * time interaction remains stable also when including interactions of time with cooperativeness of daily life interaction partners (trust) and experience in related studies.

Table S4. Tobit regression of decision times, SVO angle, cooperativeness of daily life interaction partners (trust) and experience on contributions.

Contribution (in %)	Overall Analysis	Study 1 (MTurk)	Study 2 (Lab)	Study 3 (Panel)
**Decision Time (DT in log10(sec))**	-49.05***<br>(-3.57)	-21.26<br>(-1.04)	-29.23<br>(-0.69)	-58.59**<br>(-3.17)
**Social Value Orientation (SVO angle in degree)**	2.85***<br>(10.21)	3.32***<br>(6.22)	2.71***<br>(3.85)	2.62***<br>(7.28)
**Interaction DT * SVO**	-2.31*<br>(-2.49)	-1.34<br>(-0.87)	-4.19<br>(-1.52)	-1.95<br>(-1.49)
**Trust**	5.68***<br>(4.00)	6.60**<br>(2.80)	-0.34<br>(-0.08)	6.56**<br>(3.46)
**Interaction DT * Trust**	-9.86*<br>(-1.98)	0.56<br>(0.09)	-40.28*<br>(-2.13)	-11.05<br>(-1.39)
**Experience**	-1.65<br>(-0.47)	1.39<br>(0.25)	-2.23<br>(-0.29)	3.14<br>(0.60)
**Interaction DT * Experience**	-13.70<br>(-1.30)	-20.41<br>(-1.08)	-26.68<br>(-0.84)	-15.30<br>(-0.87)
**Constant**	65.43***<br>(7.16)	41.46***<br>(6.02)	72.83***<br>(7.36)	99.80***<br>(19.27)
**Observations**	741	133	104	504
**Pseudo R^2**	0.057	0.097	0.047	0.042

+ p < .1, * p < .05, ** p < .01, *** p < .001 (two-sided)

Note. *t*-values are presented in parentheses; predictors are centered. Overall analysis also includes two study dummies which are not reported.

Persons with a high personality scores in Honesty-Humility (HH; Cronbachs alpha = .78) show a similar effect of decision time on cooperation as prosocials do, which dovetails with recent findings from mouse-tracking studies.[7]

Table S5. Tobit regression of Honesty-Humility and reaction times on contributions (Study 3)

**Contribution (in %)**	**Study 3 (Panel)**
**Decision Time (DT in log10(sec))**	-71.85** (-3.46)
**Honesty-Humility (HH)**	23.74** (3.23)
**Interaction of DT * HH**	-67.68* (-2.49)
**Constant**	102.91*** (18.07)
**Observations**	504
**Pseudo R^2**	0.010

+ p < .1, * p < .05, ** p < .01, *** p < .001 (two-sided)

Note. *t*-values are presented in parentheses; predictors are centered.

References

1 Rand, D. G., Greene, J. D. & Nowak, M. A. Spontaneous giving and calculated greed. *Nature* **489**, 427-430, doi:10.1038/nature11467 (2012).

2 Liberman, V., Samuels, S. M. & Ross, L. The name of the game: Predictive power of reputations versus situational labels in determining prisoner's dilemma game moves. *Personality and Social Psychology Bulletin* **30** 1175-1185, doi:10.1177/0146167204264004 (2004).

3 Rand, D. G., Greene, J. D. & Nowak, M. A. Spontaneous giving and calculated greed - Supplementary Information. *Nature* **489**, 1-50, doi:10.1038/nature11467 (2012).

4 Murphy, R. O., Ackermann, K. A. & Handgraaf, M. J. J. Measuring Social Value Orientation. *Judgment and Decision Making* **6**, 771–781, doi:10.2139/ssrn.1804189 (2011).

5 Oppenheimer, D. M., Meyvis, T. & Davidenko, N. Instructional manipulation checks: Detecting satisficing to increase statistical power. *Journal of Experimental Social Psychology* **45**, 867–872, doi:10.1016/j.jesp.2009.03.009 (2009).

6 Ashton, M. C. & Lee, K. Empirical, theoretical, and practical advantages of the HEXACO model of personality structure. *Personality and Social Psychology Review* **11**, 150-166, doi:10.1177/1088868306294907 (2007).

7 Kieslich, P. J. & Hilbig, B. E. Cognitive conflict in social dilemmas: An analysis of response dynamics. *Judgment and Decision Making* **9**, 510-522 (2014).
